# Prevalence of anxiety and depression symptoms in a sample of outpatients with ATTR cardiac amyloidosis

**DOI:** 10.3389/fpsyg.2022.1066224

**Published:** 2023-01-18

**Authors:** Martina Smorti, Lucia Ponti, Francesco Soffio, Alessia Argirò, Federico Perfetto, Mattia Zampieri, Carlotta Mazzoni, Alessia Tomberli, Marco Allinovi, Carlo Di Mario, Iacopo Olivotto, Francesco Cappelli

**Affiliations:** ^1^Department of Surgical, Medical and Molecular Pathology and Critical Care Medicine, University of Pisa, Pisa, Italy; ^2^Department of Humanities, University of Urbino, Urbino, Italy; ^3^Cardiomyopathy Unit, Careggi University Hospital, Florence, Italy; ^4^Tuscan Regional Amyloidosis Center, Careggi University Hospital, Florence, Italy; ^5^IV Internal Medicine Division, Careggi University Hospital, Florence, Italy; ^6^Division of Interventional Structural Cardiology, Department of Cardiothoracovascular, Careggi University Hospital, Florence, Italy

**Keywords:** cardiac amyloidosis, psychological well-being, anxiety, depression, clinical characteristics, sociodemographic characteristics

## Abstract

Patients with ATTR cardiac amyloidosis (ATTR-CA) face rare disease that could negatively influence psychological well-being with consequences on the course of the disease and quality of life. However, to date, no study analyzed the prevalence of anxiety and depression in patients with ATTR-CA and which clinical and sociodemographic characteristics are linked with these psychopathological conditions. A total of 109 consecutive patients (83% males) aged 62–90 years with ATTR-CA were recruited. In order to better understand the prevalence of anxiety and depression in ATTR-CA, a control group composed by 33 individuals equaling gender, education, and age were recruited. The level of anxiety and depression was measured using the Italian version of the Hospital Anxiety and Depression Scale (HADS). Sociodemographic and clinic characteristics were registered. Almost half of patients (49%) reported a clinical level of depression or anxiety, or both. ATTR-CA patients reported higher levels of anxiety and depression than control group. Results showed that older patients with ATTR-CA, especially females, with more advanced disease could be more at risk to develop an anxious disorder. Furthermore, being a woman, and presenting with a greater severity of symptoms, would appear to be a risk factor for developing a depressive disorder. Overall, these results highlighted the high presence of anxiety and depression in ATTR-CA patients, suggesting to physicians to pay attention to the psychological well-being of ATTR-CA patients. In fact, a psychological support for patients with high level of psychopathological disease could reduce disease burden and improve quality of life in ATTR-CA population.

## 1. Introduction

Several works have shown an increased prevalence of depression and anxiety in adults with different types of cardiovascular diseases (CVD) compared to people free from these conditions (Ormel et al., 2007; Knapp et al., 2020).

Despite the wide literature on the role of anxiety and depression on CVD, little research has investigated anxiety and depression in Transthyretin Cardiac Amyloidosis (ATTR-CA). ATTR-CA is a myocardial disease characterized by a pathological process of TTR derived amyloid deposition in the extracellular space, resulting in a progressive deterioration of cardiac function (Agha et al., 2018).

Although previous studies suggest that ATTR-CA patients reported anxious and depressive symptoms and that, in some cases, they are clinical (Stewart et al., 2018), no study has been conducted to analyze the prevalence of anxiety and depression in ATTR-CA and if clinical and sociodemographic risk factors are associated with anxiety and depression in patients with ATTR-CA.

Previous studies conducted in AL amyloidosis, a disease with a different systemic involvement, age of onset, and therapeutic approach, showed that anxiety and depression were common, reported in approximately 30% of subjects (Smorti et al., 2012, 2014, 2016; Lo et al., 2015; Shu et al., 2016; Lopes et al., 2018). Moreover, Smorti et al. found that time since the onset of cardiac symptoms was a positive predictor of anxiety in AL patients, whereas only the severity of cardiac symptoms was for depression (Smorti et al., 2012).

Our study aim is to fill the knowledge gap in ATTR-CA comprehensively analyzing the socio-demographic and clinical risk factors that may be associated with the presence of clinically significant levels of anxious and depressive symptoms in ATTR-CA patients.

## 2. Materials and methods

### 2.1. Participants and procedures

A total of 109 consecutive patients (90 males and 19 females) aged 62–90 years (M = 79.07; DS = 6.19) with ATTR-CA followed at the Tuscan Regional Amyloidosis Center in Florence, Italy, were recruited for the present study between September 2021 and June 2022. About type of ATTR-CA, 88.1% of patients were diagnosed with ATTR wild type (ATTRwt) and 11.9% with ATTR variant (ATTRv) with prevalent cardiac phenotype. All patients enrolled had a definite diagnosis and at the time of communication of the diagnosis, or of at the first evaluation in your center of a previously diagnosed patient in case of diagnosis made at another center, the same protocol is applied for all patients of our center. Each patient receives accurate counseling on the characteristics of the disease, prognosis, therapeutic possibilities, and any repercussions on family members in the event of a diagnosis of ATTR-CAv. For that reason, all patients have similar background in terms of knowledge about their disease. Moreover, with all patients, at the time of communication of the diagnosis, are discussed the pharmacological options with market access or involvement in clinical trials. However, it should be considered that to date in Italy, the only disease modified drug for ATTR-CA is Tafamidis that was refundable since the end of January 2022, therefore, patients enrolled in the study were not in treatment.

Inclusion criteria were: diagnosis of ATTR-CA according to standard international criteria (Garcia-Pavia et al., 2021), absence of cognitive impairment, and able to understand Italian language. Exclusion criteria was the presence of actual diagnosis of anxiety or depression with ongoing psychopharmacologic treatment. Only patients corresponding to the inclusion criteria were invited to participate. All contacted patients agreed to participate. Data collection was conducted by a trained psychologist who administered the questionnaire during a routine cardiological assessment. A small group of individuals without cardiac pathology balanced for gender and age (23 males; M_age_ = 76.85; SD = 5.97) were recruited from local community centers in order to compare the level of anxiety and depression between them and our ATTR-CA patients. The study was approved by the local Ethical Committee (CEAVC; protocol number 19476_OSS/2021). Participation to the study was voluntary and written informed consent was obtained from all patients before data collection by a study responsible who informed them about aims and procedure of the study.

### 2.2. Instruments and detected variables

#### 2.2.1. Socio-demographic characteristics

All participants completed a questionnaire to collect socio-demographic data, such as age, gender, educational level, marital status, have children or not, working activity, and living condition.

#### 2.2.2. Clinical characteristics

Only for ATTR-CA patients, Physicians recorded the following clinical data during cardiological assessment: Body Mass index (BMI), type of ATTR, months since communication of diagnosis, symptom severity according to NYHA class, the level of NT-proBNP, the glomerular filtration rate (GFR), interventricular septum (IVS), and Left Ventricular End Diastolic Diameter (LVEDD); LV posterior Wall (LVPW), Left Atrium Diastolic Diameter (LADD), Left Ventricular Ejection Fraction (LVEF),E/e’, and National Amyloid Center (NAC) score as previously described (Gillmore et al., 2018; Cappelli et al., 2020), and medical treatment.

#### 2.2.3. Psychopathological characteristics

The Italian version of the Hospital Anxiety and Depression Scale (HADS; Iani et al., 2014) was employed to assess the level of anxious and depressive symptoms. The HADS is a self-reported questionnaire composed by 14 items (seven assessing anxiety and seven assessing depression) rated on a four-point Likert scale from 0 to 3. Scores range from 0 to 21 for each subscale, with a higher score indicating a higher level of psychological symptomatology. In the present study, the HADS Cronbach’s alpha was 0.86 and 0.81 for the anxiety and depression subscales, respectively.

### 2.3. Data analyses

The prevalence of anxiety and depression symptoms were estimated using the HADS cut-off points of 8. Based on score 8 on anxiety or depression HADS subscales participants with a clinical level of psychopathological symptomatology were identified. Comparison between ATTR-CA patients and control group was performed. Differences in presence of clinical level of psychopathological symptomatology according to socio-demographic and clinical variables were performed using chi-square analysis and Student’s *t*-test, depending on the dichotomous or continuous nature of variables. Two regression analyses were performed to verify how the different significant variables were associated with anxiety and depression. All comparisons were calculated in IBM SPSS Statistics for Macintosh, Version 23.0 (IBM, Armonk, NY, United States), and *p* < 0.05 was considered significant.

## 3. Results

Socio-demographic and clinical characteristics of patients and control group are reported in Supplementary Table 1. As expected, most patients were male with an average age of almost 80 years, they were married, lived with their partner, and had at least one child. All patients were retired. Moreover, most of them had wild type ATTR-CA and they had been diagnosed for an average of 2 years before recruitment. At study, evaluation 19% of patients were in NYHA class I, 66% in NYHA class II, and 15% in NYHA class III. Echocardiographic evaluation performed at the moment of recruitment showed a slight reduction in left ventricular systolic function, and impaired left ventricular relaxation measured by the E/e’ ratio (Supplementary Table 1). No significant differences emerged between ATTR-CA patients and control group on the socio-demographical variables.

**Figure 1:**
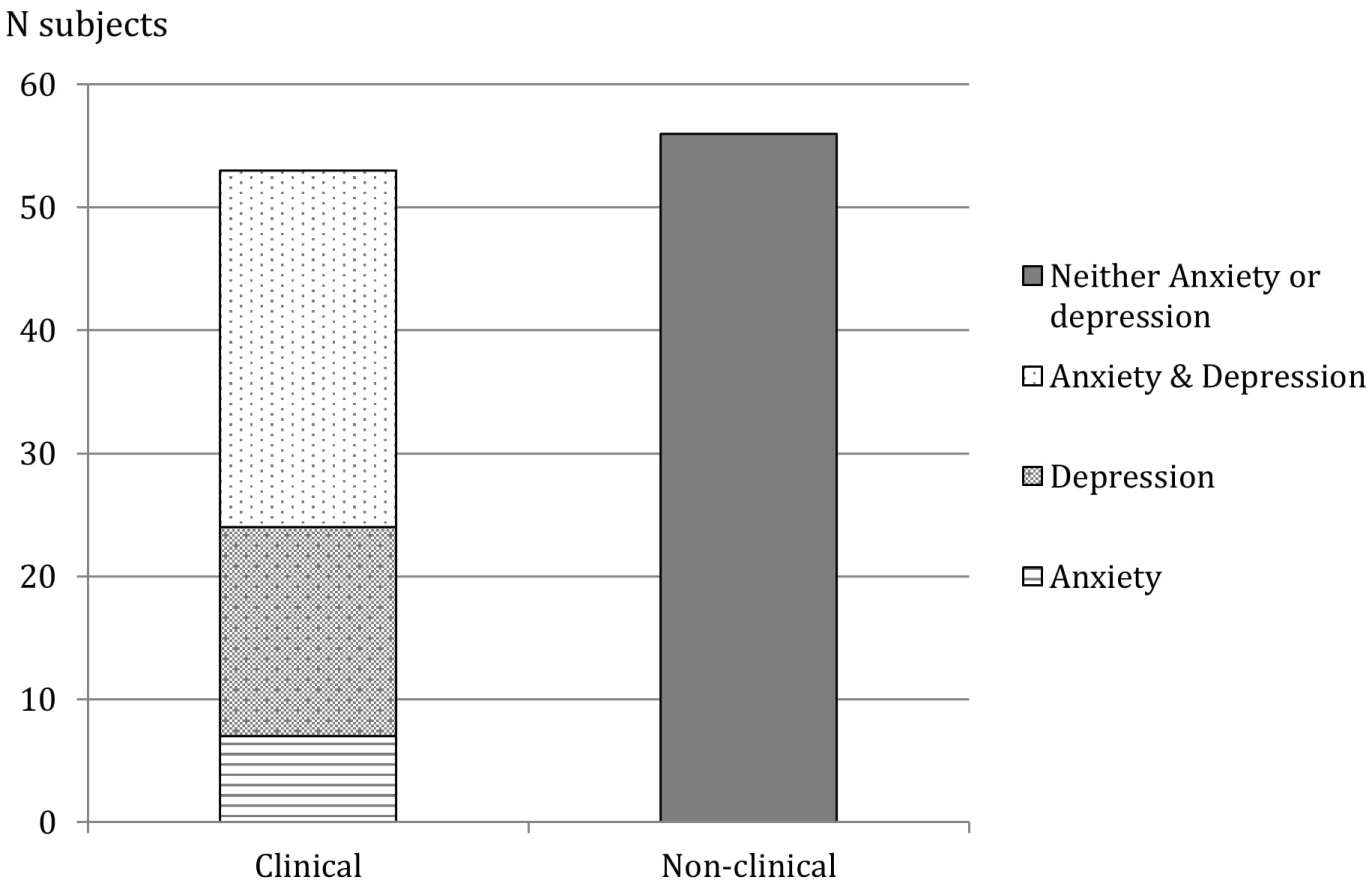
Clinical and non-clinical subjects according to HADS. Prevalence of anxiety and depression and both anxiety and depression are reported.

Overall, 53 patients (48.6%) reported a clinical level of depression or anxiety, or both (Figure 1).

Taking in consideration anxiety and depression separately (and not comorbid), 36 patients (33%) meet criteria for clinical level of anxiety and 46 (42.2%) for depression. Among participant of control group, 5 of theme (15.2%) reported significant level of anxious and 7 (21.2%) of depressive symptoms. Moreover, ATTR-CA patients reported higher levels of anxiety and depression than control group.

Referring to anxious symptomatology, no significant differences emerged between anxious and not anxious patients on most socio-demographical and clinical characteristics excepted for gender, females reported more likely a clinical presence of anxiety than males (69 vs. 31%, *p* = 0.011); age, with patients with a clinical level of anxiety presented an older age (*p* = 0.047); and renal function impairment, with patients with a clinical level of anxiety showing a lower GFR (*p* = 0.021). Similar results are highlighted referring to depressive symptomatology. According to clinical depression levels, patients differed on gender, females reported more likely a clinical presence of depression than males (74 vs. 36%, *p* = 0.042); NYHA class, where patients in class III reported more likely the presence of clinical level of depression (*p* = 0.015), and NAC score, with a lower prevalence of patient with clinical level of depression in class 1 (*p* = 0.05). All these results are reported in Supplementary Table 2.

Finally, results of regression analysis conducted using anxiety as dependent variables and age, gender and GFR level as predictors, showed that only gender was significantly associated to the level of anxiety. The regression analysis conducted using depression as dependent variables and gender, NYHA class, and NAC score and predictors showed that female gender and higher NYHA class were significantly associated to the level of depression (see Supplementary Table 3).

## 4. Discussion

ATTR cardiac amyloidosis is a rare disease associated to a high mortality rate (Rapezzi et al., 2013; González-López et al., 2017), and the incidence of this disease is uniformly considered to increase. This is presumably due to better knowledge of the disease itself and the availability of imaging tests that can reveal its presence (Zampieri et al., 2021). Moreover, as many chronic diseases, with poor outcome, it can easily be accompanied by tiredness, pain, discomfort, anxious, and depressive feelings (Damy et al., 2022). An evaluation of the psychological burden should be considered mandatory due to the influences that a clinically significant psychopathological condition can have on the patient’s well-being and on the course of his/her illness (Gathright et al., 2017). Several studies showed that ATTR-CA is linked to high levels of impairment in different domains, including physical health, quality of life, and reduced productivity (Stewart et al., 2018; Aimo et al., 2021). It is possible to hypothesize that the high levels of anxiety and depression can significantly and negatively influence all these aspects. Despite that, to our knowledge, no studies have investigated the prevalence of anxiety and depression in ATTR-CA patients, exploring which variables, both socio-demographic and clinical, may be most associated with these conditions. This knowledge could instead represent a useful tool for physicians who work with these patients. Knowing which aspects are most associated with psychopathological traits, such as anxiety and depression, could allow physicians to identify possible risk situations more quickly and promptly arrange a specific support. At this regard, the purpose of this study is therefore aimed at filling this aspect. Firstly, our results confirmed that depression and anxiety are prevalent among ATTR-CA patients, with almost half of patients (49%) reported a clinical level of depressive or anxious symptoms, or both. ATTR-v, given its nature as an inherited disease, may be associated with the development of feelings of guilt related to the possibility of having passed the genetic mutation to offspring (Stewart et al., 2018). Nevertheless, this study found that anxiety and depressive symptoms were not more prevalent in the subgroup of ATTR-v patients than those with ATTR-wt. However, considering that most patients had some form of ATTR-wt, caution is needed in interpreting these results and further investigation would be needed.

Comparing the prevalence of anxious and depressive symptoms with those reported in the general population aged 65–80 years, the prevalence of anxious symptoms among ATTR-CA is 3-fold higher (33 vs. 10% in the general population), and that of depressive symptoms almost 4-fold higher (42 vs. 9%) than general population (Djukanovic et al., 2017). Also in the present study, the levels of anxious and depressive symptoms in ATTR-CA patients were higher than in control group. Nevertheless, the prevalence of symptoms of anxiety and depression was comparable to that found in other cardiomyopathy (Singh et al., 2021). Moreover, it should be noted that our study was conducted during the COVID-19 pandemic, when the levels of symptoms of anxiety and depression increased in the general Italian population (and in the elderly) compared to the pre-COVID period (Fiorenzato et al., 2021).

This high prevalence highlights the need to pay particular attention to the anxiety and depression levels of ATTR-CA patients. Referring to anxiety, our results showed that older patients, especially females, with more advanced disease (suggested by lower GFR levels often linked to a cardiorenal syndrome) could be more at risk to develop an anxious disorder. Being a woman is also a risk factor for depressive disorder, along with greater symptom severity, as measured by the NYHA class scale and again the disease severity stage according to the NAC score. Although these data are significant results, it should be noted that the group is strongly unbalanced with respect to gender. This reflects the gender distribution within the ATTR-CA population, which predominantly affects the male population (Grogan et al., 2016). However, the low number of women in the sample must be cautious in the possibility of generalizing these results. Moreover, considering all significant variables, referring to anxiety symptoms, only gender was a significant predictor, while for depression symptoms both gender and NYHA class are predictors.

Unfortunately, all the other variables taken into consideration, both socio-demographic and clinic, did not show any significant association with the presence of clinical level of anxious and depressive symptoms. This does not allow us to identify a specific socio-demographic profile of a patient at risk of psychopathology, with particular characteristics, beyond gender and the above mentioned few other clinical indicators. These results are however very important, because they underline the high presence of clinical symptoms of anxiety and depression in the population of ATTR-CA patients, suggesting to pay attention, in general, to all patients followed for this disease.

In other words, our data seem suggest the relevance in amyloid referral centers a specific psychological assessment performed by trained health care staff should be provided to entire patient population. In fact, identify those subjects with psychopathological difficulties should be allow to refer them for further evaluation and take in charge by psychological staff. At our center, for example, for patients who scored clinical symptoms of anxiety and depression, our psychologist conducted a return to each patient of the questionnaire result and offered the opportunity to have a psychological support interview at the center itself. A psychological support could be able to reduce disease burden and improve quality of life and adherence to new disease modifying drugs that are changing the scenario of ATTR-CA.

### 4.1. Limitations

There are some study limitations. Firstly, the sample is small, it is possible that our results could be susceptible to type II error. However, ATTR-CA is a rare chronic disease and this study had a monocentric nature. This is undoubtedly another limitation. Due this monocentric nature this data should be confirmed in larger and multicentric, international population to avoid referral center bias. Nevertheless, given the lack of studies on this topic, the present study represents a starting point contributing to the literature on the prevalence of anxious and depressive symptoms in ATTR-CA population.

Another limitation is linked to the lack of other information which could influence the presence of anxious and depressive symptoms, such as the presence or number of hospitalizations, the socio-economic status, or a baseline psychological status before the diagnosis. Moreover, in the present study the presence of anxiety and depression was assessed by a self-report questionnaire. There is no doubt that a clinical evaluation carried out by a specialist would be more precise and reliable. However, our study was not intended to make a psychopathological diagnosis according to the DSM-5 criteria (American Psychiatric Association, 2014), but only to detect depressive or anxious symptoms above the cut off in our sample of patients with ATTR-CA, in order to highlight whether psychological distress was particularly presented in this clinical population. Moreover, the HADS is a valid measure for detecting the presence of anxious and depressive symptoms, widely used in hospitals and adapted to various medical diseases including cardiomyopathies. Furthermore, compared to other self-report assessment tools, the items have been created in a way to avoid ambiguous somatic symptoms that can be associated with various medical conditions, such as dizziness and lethargy (Zigmond & Snaith, 1983).

In conclusion, ATTR-CA is a debilitating disease not only physically but could also constitute a risk factor with respect to psychological well-being. In fact, despite the limitations of the present study, the results suggest the need to pay attention to the presence of anxiety and depression in this population. There is a need for increased awareness among the medical community about the prevalence of psychological disorders to provide a psychological assessment as soon as patients are referred to specialized centers for a diagnosis. In fact, since it is currently not possible to provide a specific profile of ATTR-CA patients with a higher risk of suffering from anxious or depressive symptoms, a specific psychological evaluation performed by healthcare professionals should be carried out in the amyloid reference centers trained on the entire patient population.

## Data availability statement

The raw data supporting the conclusions of this article will be made available by the authors, without undue reservation.

## Ethics statement

The studies involving human participants were reviewed and approved by Comitato Etico Area Vasta Centro (CEAVC). The patients/participants provided their written informed consent to participate in this study.

## Author contributions

MS contributed to the literature review, drafting, and revising the article and approval of the final version. LP also contributed to the literature review and drafting, design, and revision of the article. FS contributed to the literature review and drafting of the article. AA, MZ, and CM contributed in acquisition of data. AT contributed in data analysis. FP and MA contributed in data interpretation. CDM, IO, and FC conceived the study and reviewed and approved the final manuscript. All authors contributed to the article and approved the submitted version.

## Conflict of interest

The authors declare that the research was conducted in the absence of any commercial or financial relationships that could be construed as a potential conflict of interest.

## Publisher’s note

All claims expressed in this article are solely those of the authors and do not necessarily represent those of their affiliated organizations, or those of the publisher, the editors and the reviewers. Any product that may be evaluated in this article, or claim that may be made by its manufacturer, is not guaranteed or endorsed by the publisher.

## References

[ref1] Agha A. M. Parwani P. Guha A. Durand J. B. Iliescu C. A. Hassan S.. (2018). Role of cardiovascular imaging for the diagnosis and prognosis of cardiac amyloidosis. Open Heart 5:e000881. doi: 10.1136/openhrt-2018-000881 30305910 PMC6173267

[ref2] Aimo A. Rapezzi C. Perfetto F. Cappelli F. Palladini G. Obici L.. (2021). Quality of life assessment in amyloid transthyretin (ATTR) amyloidosis. Eur. J. Clin. Investig. 51:e13598. doi: 10.1111/eci.13598, PMID: 33982288 PMC8596396

[ref3] American Psychiatric Association (2014). DSM-5. Manuale Diagnostico e Statistico dei Disturbi Mentali. Milano: Cortina Raffaello.

[ref4] Cappelli F. Martone R. Gabriele M. Taborchi G. Morini S. Vignini E.. (2020). Biomarkers and prediction of prognosis in Transthyretin-related cardiac amyloidosis: direct comparison of two staging systems. Can. J. Cardiol. 36, 424–431. doi: 10.1016/j.cjca.2019.12.020, PMID: 32145869

[ref5] Damy T. Adams D. Bridoux F. Grateau G. Planté-Bordeneuve V. Ghiron Y.. (2022). Amyloidosis from the patient perspective: the French daily impact of amyloidosis study. Amyloid 29, 165–174. doi: 10.1080/13506129.2022.2035354, PMID: 35144512

[ref6] Djukanovic I. Carlsson J. Årestedt K. (2017). Is the hospital anxiety and depression scale (HADS) a valid measure in a general population 65–80 years old? A psychometric evaluation study. Health Qual. Life Outcomes 15, 1–10. doi: 10.1186/s12955-017-0759-9, PMID: 28978356 PMC5628437

[ref7] Fiorenzato E. Zabberoni S. Costa A. Cona G. (2021). Cognitive and mental health changes and their vulnerability factors related to COVID-19 lockdown in Italy. PLoS One 16:e0246204. doi: 10.1371/journal.pone.0246204, PMID: 33503055 PMC7840042

[ref8] Garcia-Pavia P. Rapezzi C. Adler Y. Arad M. Basso C. Brucato A.. (2021). Diagnosis and treatment of cardiac amyloidosis: a position statement of the ESC working group on myocardial and pericardial diseases. Eur. Heart J. Cardiovasc. Pharmacother. 42, 1554–1568. doi: 10.1093/eurheartj/ehab072, PMID: 33825853 PMC8060056

[ref9] Gathright E. C. Goldstein C. M. Josephson R. A. Hughes J. W. (2017). Depression increases the risk of mortality in patients with heart failure: a meta-analysis. J. Psychosom. Res. 94, 82–89. doi: 10.1016/j.jpsychores.2017.01.010, PMID: 28183407 PMC5370194

[ref10] Gillmore J. D. Damy T. Fontana M. Hutchinson M. Lachmann H. J. Martinez-Naharro A.. (2018). A new staging system for cardiac transthyretin amyloidosis. Eur. Heart J. 39, 2799–2806. doi: 10.1093/eurheartj/ehx589, PMID: 29048471

[ref11] González-López E. Gagliardi C. Dominguez F. Quarta C. C. de Haro-del Moral F. J. Milandri A.. (2017). Clinical characteristics of wild-type transthyretin cardiac amyloidosis: disproving myths. Eur. Heart J. 38, 1895–1904. doi: 10.1093/eurheartj/ehx043, PMID: 28329248

[ref12] Grogan M. Scott C. G. Kyle R. A. Zeldenrust S. R. Gertz M. A. Lin G.. (2016). Natural history of wild-type transthyretin cardiac amyloidosis and risk stratification using a novel staging system. J. Am. Coll. Cardiol. 68, 1014–1020. doi: 10.1016/j.jacc.2016.06.033, PMID: 27585505

[ref13] Iani L. Lauriola M. Costantini M. A. (2014). Confirmatory bifactor analysis of the hospital anxiety and depression scale in an Italian community sample. Health Qual. Life Outcomes 12, 1–8. doi: 10.1186/1477-7525-12-84, PMID: 24902622 PMC4054905

[ref14] Knapp P. Dunn-Roberts A. Sahib N. Cook L. Astin F. Kontou E.. (2020). Frequency of anxiety after stroke: an updated systematic review and meta-analysis of observational studies. Int. J. Stroke 15, 244–255. doi: 10.1177/1747493019896958, PMID: 31980004

[ref15] Lo S. Shu J. Phillips M. Sun F. Berk J. L. Sanchorawala V. (2015). Symptoms of depression and anxiety assessed by the SF-36 questionnaire in patients with AL amyloidosis. Blood 126:3299. doi: 10.1182/blood.V126.23.3299.3299 27460276

[ref16] Lopes A. Fonseca I. Sousa A. Rodrigues C. Branco M. Coelho T.. (2018). Psychopathological dimensions in subjects with hereditary ATTR V30M amyloidosis and their relation with life events due to the disease. Amyloid 25, 26–36. doi: 10.1080/13506129.2018.1428795, PMID: 29357699

[ref17] Ormel J. Von Korff M. Burger H. Scott K. Demyttenaere K. Huang Y.. (2007). Mental disorders among persons with heart disease—results from world mental health surveys. Gen. Hosp. Psychiatry 29, 325–334. doi: 10.1016/j.genhosppsych.2007.03.009, PMID: 17591509 PMC2048744

[ref18] Rapezzi C. Quarta C. C. Obici L. Perfetto F. Longhi S. Salvi F.. (2013). Disease profile and differential diagnosis of hereditary transthyretin-related amyloidosis with exclusively cardiac phenotype: an Italian perspective. Eur. Heart J. 34, 520–528. doi: 10.1093/eurheartj/ehs123, PMID: 22745357

[ref19] Shu J. Lo S. Phillips M. Sun F. Seldin D. C. Berenbaum I.. (2016). Depression and anxiety in patients with AL amyloidosis as assessed by the SF-36 questionnaire: experience in 1226 patients. Amyloid 23, 188–193. doi: 10.1080/13506129.2016.1208081, PMID: 27460276

[ref20] Singh S. M. Murray B. Tichnell C. McClellan R. James C. A. Barth A. S. (2021). Anxiety and depression in inherited channelopathy patients with implantable cardioverter-defibrillators. Heart Rhythm. 2, 388–393. doi: 10.1016/j.hroo.2021.06.001, PMID: 34430944 PMC8369306

[ref21] Smorti M. Cappelli F. Bergesio F. Perfetto F. (2012). Anxiety and depression among AL amyloidosis patients: the role of cardiac symptoms. Amyloid 19, 123–128. doi: 10.3109/13506129.2012.687420, PMID: 22624655

[ref22] Smorti M. Cappelli F. Guarnieri S. Bergesio F. Perfetto F. (2014). Depression and cardiac symptoms among AL amyloidosis patients: the mediating role of coping strategies. Psychol. Health Med. 19, 263–272. doi: 10.1080/13548506.2013.802357, PMID: 23725340

[ref23] Smorti M. Guarnieri S. Bergesio F. Perfetto F. Cappelli F. (2016). Anxiety and depression among amyloid light-chain cardiac amyloidosis patients: the role of life satisfaction. Eur. J. Cardiovasc. Nurs. 15, 269–275. doi: 10.1177/1474515114566737, PMID: 25601945

[ref24] Stewart M. Shaffer S. Murphy B. Loftus J. Alvir J. Cicchetti M.. (2018). Characterizing the high disease burden of transthyretin amyloidosis for patients and caregivers. Neurol. Therapy 7, 349–364. doi: 10.1007/s40120-018-0106-z, PMID: 30073497 PMC6283802

[ref25] Zampieri M. Nardi G. Del Monaco G. Allinovi M. Gabriele M. Zocchi C.. (2021). Changes in the perceived epidemiology of amyloidosis: 20 year-experience from a tertiary referral Centre in Tuscany. Int. J. Cardiol. 335, 123–127. doi: 10.1016/j.ijcard.2021.04.023, PMID: 33865873

[ref26] Zigmond A. S. Snaith R. P. (1983). The hospital anxiety and depression scale. Acta Psychiatr. Scand. 67, 361–370. doi: 10.1111/j.1600-0447.1983.tb09716.x 6880820

