# Supplementary material for: Prevalence of anxiety and depression symptoms in a sample of outpatients with ATTR cardiac amyloidosis
Source: Front Psychol. 2023 Jan 18;13:1066224. doi: 10.3389/fpsyg.2022.1066224 (PMC9889831; doi:10.3389/fpsyg.2022.1066224)
Supplement: Supplementary file 1 [file Data_Sheet_1.docx]

Supplementary Material

# Supplementary Figures and Tables

Table 1. Characteristics of study sample

| **Characteristics** | **ATTR-CA patients** | **Control group** | **χ^2^ (df)** | **t(140)** | **p** |
| --- | --- | --- | --- | --- | --- |
|  |  |  |  |  |  |
| Age in years, mean (SD) | 79.07 (6.19) | 76.85 (5.97) |  | 1.822 | .071 |
| Male, n (%) | 90 (82.6) | 23 (69.7) | 2.58(1) |  | .108 |
| Marital status, n (%) |  |  | 2.67(2) |  | .263 |
| Married | 81 (74.3) | 29 (87.9) |  |  |  |
| Unmarried | 7 (6.4) | 1 (3) |  |  |  |
| Widower | 21 (19.3) | 3 (9.1) |  |  |  |
| Children, n (%) |  |  |  |  |  |
| Yes | 95 (87.2) | 32 (97) | 2.58(1) |  | .108 |
| No | 14 (12.8) | 1 (3) |  |  |  |
| Living condition, n (%) |  |  | 1.55(2) |  | .461 |
| Lives alone | 28 (25.7) | 6 (18.2) |  |  |  |
| Lives with partner | 70 (64.2) | 25 (75.8) |  |  |  |
| Lives with partner and children | 11 (10.1) | 2 (6) |  |  |  |
| Educational level, n (%) |  |  | 6.18(3) |  | .103 |
| Primary school | 53 (48.6) | 11 (33.3) |  |  |  |
| Secondary education | 33 (30.3) | 8 (24.2) |  |  |  |
| High school diploma | 19 (17.4) | 11 (33.3) |  |  |  |
| Bachelor’s degree | 4 (3.7) | 3 (9.1) |  |  |  |
| HADS_anxiety, mean (SD) | 6.63 (4.30) | 5.06 (2.25) |  | 2.02 | .046 |
| HADS_depression, mean (SD) | 6.99 (4.57) | 4.18 (3.18) |  | 3.29 | .001 |
| BMI, mean (SD) | 25.54 (3.31) |  |  |  |  |
| Type of ATTR, n (%) |  |  |  |  |  |
| WT | 96 (88.1) |  |  |  |  |
| Variant | 13 (11.9) |  |  |  |  |
| Ile68Leu | 10/13 |  |  |  |  |
| Glu74Gln | 1/13 |  |  |  |  |
| Phe84Ile | 1/13 |  |  |  |  |
| Val122Ile | 1/13 |  |  |  |  |
| Months since communication of diagnosis, mean (SD) | 24.45 (19.51) |  |  |  |  |
| Symptom severity, n (%) |  |  |  |  |  |
| NYHA class I | 21 (19.3) |  |  |  |  |
| NYHA class II | 72 (66.1) |  |  |  |  |
| NYHA class III | 16 (14.6) |  |  |  |  |
| NYHA class IV | 0 (0) |  |  |  |  |
| NT-proBNP, pg/ml mean (SD) | 2472.30 (2498.50) |  |  |  |  |
| GFR, ml/min/m^2^ mean (SD) | 65.53 (19.00) |  |  |  |  |
| IVS_mm, mean (SD) | 16.77 (2.57) |  |  |  |  |
| LVEDD_mm, mean (SD) | 43.93 (5.83) |  |  |  |  |
| LVPW_mm, mean (SD) | 15.00 (2.10) |  |  |  |  |
| LADD mm, mean (SD) | 45.90 (11.38) |  |  |  |  |
| EF, % mean (SD) | 55 (9) |  |  |  |  |
| E/e’, mean (SD) | 15.08 (4.72) |  |  |  |  |
| NAC_score, n (%) |  |  |  |  |  |
| Class I | 67 (61.5) |  |  |  |  |
| Class II | 30 (27.5) |  |  |  |  |
| Class III | 12 (11) |  |  |  |  |
| Anticoagulant, n (%) |  |  |  |  |  |
| Warfarin | 2 (3) |  |  |  |  |
| Doac | 58 (53) |  |  |  |  |
| Mineral corticoid receptor antagonist, n (%) | 31 (28) |  |  |  |  |
| Furosemide, n (%) |  |  |  |  |  |
| 12.5 mg | 5 (5) |  |  |  |  |
| 25 mg | 40 (37) |  |  |  |  |
| 50 mg | 13 (12) |  |  |  |  |
| 75 mg | 8 (7) |  |  |  |  |
| 120 mg | 3 (3) |  |  |  |  |
| 125 mg | 13 (12) |  |  |  |  |
| 175 mg | 5 (5) |  |  |  |  |
| 250 mg | 3 (3) |  |  |  |  |
| 500 mg | 1 (1) |  |  |  |  |
| Calcium antagonist, n (%) | 14 (13) |  |  |  |  |
| ACE/ARB, n (%) | 40 (37) |  |  |  |  |
| ARNI, n (%) | 1 (1) |  |  |  |  |
| SGLT2 inhibitors, n (%) | 6 (6) |  |  |  |  |
| Bisoprolol, n (%) |  |  |  |  |  |
| 1.25 mg | 10 (9) |  |  |  |  |
| 2.5 mg | 15 (14) |  |  |  |  |
| 5 mg | 2 (2) |  |  |  |  |
| 7.5 mg | 1 (1) |  |  |  |  |
| 10 mg | 1 (1) |  |  |  |  |
| Atenolol, n (%) |  |  |  |  |  |
| 50 mg | 1 (1) |  |  |  |  |
| 75 mg | 1 (1) |  |  |  |  |
| Carvedilol, n (%) |  |  |  |  |  |
| 25 mg | 2 (2) |  |  |  |  |
| Metoprolol, n (%) |  |  |  |  |  |
| 100 mg | 3 (3) |  |  |  |  |
| Nebivolol, n (%) |  |  |  |  |  |
| 5 mg | 4 (4) |  |  |  |  |
| Comorbidity |  |  |  |  |  |
| Coronary artery disease | 12 (11) |  |  |  |  |
| History of syncope | 9 (8) |  |  |  |  |
| Diabetes | 13 (12) |  |  |  |  |
| Hypertension | 50 (46) |  |  |  |  |
| Kidney failure | 37 (34) |  |  |  |  |
| Carpal tunnel | 15 (14) |  |  |  |  |

*Note.* NYHA, New York Heart Association; GFR, glomerular filtration rate; IVS, interventricular septum; LV, Left Ventricular; LVEDD, LV End Diastolic Diameter, LV PW, LV posterior Wall; LADD, Left Atrium Diastolic Diameter; LVEF, Left Ventricular Ejection Fraction; E/e’; NAC, National Amyloid Centre score; HADS, Hospital Anxiety and Depression Scale.

Table 2. Descriptive statistics of socio-demographic and clinical characteristics by anxious groups, and analysis results.

|  | Anxious patients (n=36) | Not anxious patients (n=73) | χ^2^ (df) | t(107) | p | Depressed patients (n=46) | Not depressed patients (n=63) | χ^2^ (df) | t(107) | P |
| --- | --- | --- | --- | --- | --- | --- | --- | --- | --- | --- |
| Age in years, mean (SD) | 80.75 (5.78) | 78.25 (6.26) | - | -2.01 | **.047** | 80.24 (5.72) | 78.22 (6.43) | - | -1.69 | .093 |
| Gender, n (%) |  |  | 6.43 (1) | - | **.011** |  |  | 4.14 (1) | - | **.042** |
| Females | 11(30.6) | 8 (11.0) |  |  |  | 12 (26.1) | 7 (11.1) |  |  |  |
| Males | 25 (69.4) | 65 (89.0) |  |  |  | 34 (73.9) | 56 (88.9) |  |  |  |
| Marital status, n (%) |  |  | 1.34 (2) | - | .507 |  |  | .33 (2) | - | .850 |
| Married | 27 (75.0) | 54 (74.0) |  |  |  | 33 (71.7) | 48 (76.2) |  |  |  |
| Unmarried | 1 (2.8) | 6 (8.2) |  |  |  | 3 (6.5) | 4 (6.3) |  |  |  |
| Widower | 8 (22.2) | 13 (17.8) |  |  |  | 10 (21.7) | 11 (17.5) |  |  |  |
| Children n (%) |  |  | 2.55 (1) | - | .110 |  |  | .28 (1) | - | .599 |
| Yes | 34 (94.4) | 61 (83.6) |  |  |  | 41 (89.1) | 54 (85.7) |  |  |  |
| No | 2 (5.6) | 12 (16.4) |  |  |  | 5 (10.9) | 9 (14.3) |  |  |  |
| Living condition n (%) |  |  | .23 (3) | - | .894 |  |  | 3.01 (2) | - | .222 |
| Lives alone | 9 (25.0) | 19 (26.0) |  |  |  | 13 (28.3) | 15 (23.8) |  |  |  |
| Lives with partner | 24 (66.7) | 46 (63.0) |  |  |  | 26 (56.5) | 44 (69.8) |  |  |  |
| Lives with partner and children | 3 (8.3) | 8 (11.0) |  |  |  | 7 (15.2) | 4 (6.3) |  |  |  |
| Educational level, n (%) |  |  | 1.28 (3) |  | .515 |  |  | 3.10 (3) |  | .377 |
| Primary school | 17 (47.3) | 36 (49.3) |  |  |  | 23 (50.0) | 30 (47.6) |  |  |  |
| Secondary education | 12 (33.3) | 21 (28.8) |  |  |  | 15 (32.6) | 18 (28.6) |  |  |  |
| High school diploma | 7 (19.4) | 12 (16.4) |  |  |  | 8 (17.4) | 11 (17.5) |  |  |  |
| Bachelor’s degree | - | 4 (5.5) |  |  |  | - | 4 (6.3) |  |  |  |
| BMI | 25.36 (3.63) | 25.40 (3.13) |  | 054 | .957 | 25.20 (3.36) | 25.52 (3.25) |  | .513 | .609 |
| Type of ATTR |  |  | 2.08 (1) | - | .150 |  |  | .10 (1) | - | .759 |
| WT n (%) | 34 (94.4) | 62 (84.9) |  |  |  | 40 (87.0) | 56 (88.9) |  |  |  |
| Variant n (%) | 2 (5.6) | 11 (15.1) |  |  |  | 6 (13.0) | 7 (11.1) |  |  |  |
| Months since communication of diagnosis, mean (SD) | 23.00 (16.91) | 25.16 (20.75) | - | .54 | .588 | 26.87 (22.74) | 22.68 (16.73) | - | -1.11 | .270 |
| Symptom severity |  |  | 3.94 (2) | - | .139 |  |  | 8.39 (2) |  | .**015** |
| NYHA class I, n (%) | 4 (11.1) | 17 (23.3) |  |  |  | 7 (15.2) | 14 (22.2) |  |  |  |
| NYHA class II, n (%) | 24 (66.7) | 48 (65.8) |  |  |  | 27 (58.7) | 45 (71.4) |  |  |  |
| NYHA class III, n (%) | 8 (22.2) | 8 (11.0) |  |  |  | 12 (26.1) | 4 (6.3) |  |  |  |
| NYHA class IV, n (%) | - | - |  |  |  | - | - |  |  |  |
| NT-proBNP, pg/ml mean (SD) | 2628.53 (2673.36) | 2395.26 (2423.08) | - | -.46 | .649 | 2967.67 (2783.69) | 2110.60 (2221.43) | - | -1.79 | .077 |
| GFR, ml/min/m^2^ mean (SD) | 57.59 (17.20) | 66.43 (19.28) | - | 2.34 | **.021** | 60.50 (18.56) | 65.75 (19.16) | - | 1.43 | .155 |
| IVS_mm, mean (SD) | 16.58 (2.41) | 16.86 (2.41) | - | .53 | .595 | 17.17 (2.47) | 16.48 (2.61) | - | -1.41 | .162 |
| LVEDD_mm, mean (SD) | 44.47 (5.84) | 43.66 (5.85) | - | -.68 | .495 | 43.76 (5.96) | 44.05 (5.78) | - | .25 | .801 |
| LVPW_mm, mean (SD) | 14.92 (1.83) | 15.04 (2.23) | - | .29 | .773 | 15.24 (1.90) | 14.83 (2.23) | - | -1.02 | .312 |
| LADD mm mean (SD) | 45.17 (5.77) | 46.36 (13.21) | - | .52 | .607 | 45.20 (7.90) | 46.52 (13.26) | - | .61 | .546 |
| EF, % mean (SD) | .54 (.10) | .55 (.09) | - | .81 | .422 | .53 (.10) | .56 (.08) | - | -1.86 | .066 |
| E/e’, mean (SD) | 15.14 (4.79) | 15.05 (4.72) | - | .69 | .688 | 16.00 (5.20) | 14.41 (4.25) | - | -1.75 | .083 |
| NAC_score, n (%) |  |  | 2.49 (2) | - | .288 |  |  | 6.00 (2) |  | **.050** |
| Class I, n (%) | 15 (46.9) | 42 (63.6) |  |  |  | 18 (45.0) | 39 (67.2) |  |  |  |
| Class II, n (%) | 12 (37.5) | 17 (25.8) |  |  |  | 14 (35.0) | 15 (25.9) |  |  |  |
| Class III, n (%) | 5 (15.6) | 7 (10.6) |  |  |  | 8 (20.0) | 4 (6.9) |  |  |  |

Table 3. Summary of the two linear regression analyses predicting anxiety and depression

|  | Anxiety | | | |
| --- | --- | --- | --- | --- |
|  | ß | t | p | 95% CI |
| Age | .15 | 1.53 | .130 | -.031 .241 |
| Gender | .23 | 2.432 | .017 | .474 4.661 |
| GFR level | -.08 | -.848 | .398 | -.064 .026 |
|  | Depression | | | |
| Gender | .20 | 2.254 | .026 | .294 4.585 |
| NYHA class | .273 | 2.857 | .005 | .655 3.623 |
| NAC score | .125 | 1.312 | .192 | -.426 2.090 |
